# Combining 2D Planimetry and Yeo’s Index Can Help Accurately Identify Patients with Severe Rheumatic Mitral Stenosis—A Perspective from a 3D Assessment Using Transoesophageal Echocardiography

**DOI:** 10.3390/diagnostics14131440

**Published:** 2024-07-05

**Authors:** Tony Li, Ryan Leow, Meei Wah Chan, William K. F. Kong, Ivandito Kuntjoro, Kian Keong Poh, Ching Hui Sia, Tiong Cheng Yeo

**Affiliations:** 1 Department of Cardiology, National University Heart Centre Singapore, Singapore 119228, Singapore; 2 Department of Medicine, Yong Loo Lin School of Medicine, National University of Singapore, Singapore 119077, Singapore

**Keywords:** mitral stenosis, rheumatic heart disease, transesophageal echocardiogram, pressure half-time, net atrioventricular compliance

## Abstract

Background: Yeo’s index is a novel measure of the severity of rheumatic mitral valve stenosis (MS). It is derived from the product of the mitral leaflet separation index and dimensionless index. This study aims to validate Yeo’s index using a transesophageal echocardiogram (TEE) three-dimensional (3D) mitral valve area (MVA) as a comparator and to compare the concordance of existing echocardiographic measures of the MVA with TEE 3DMVA. Methods and Results: We studied 111 patients with rheumatic MS who underwent both transthoracic echocardiography (TTE) and a TEE assessment of MS severity. Yeo’s index, the MVA determined by 2D planimetry, pressure half-time (PHT) and continuity equation (CE) measured on TTE were compared with the TEE 3DMVA. With a linear correlation, Yeo’s index showed the best correlation with TEE 3DMVA (r^2^ = 0.775), followed by 2D planimetry (r^2^ = 0.687), CE (r^2^ = 0.598) and PHT (r^2^ = 0.363). Using TEE 3DMVA as comparator, Yeo’s index (*ρ*_c_ = 0.739) demonstrated the best concordance, followed by 2D planimetry (*ρ*_c_ = 0.632), CE (*ρ*_c_ = 0.464) and PHT (*ρ*_c_ = 0.366). When both Yeo’s index and 2D planimetry suggested significant MS, the positive predictive value was high (an AUC of 0.966 and a PPV of 100.00% for severe MS, and an AUC of 0.864 and a PPV of 85.71% for very severe MS). When both measures suggested the absence of significant MS, the negative predictive value was also high (an AUC of 0.940 and an NPV of 88.90% for severe MS, and an AUC of 0.831 and an NPV of 88.71% for very severe MS). Conclusions: Yeo’s index performed well in identifying severe MS when compared with TEE 3DMVA and may be a useful adjunct to existing methods of measuring MS severity. Combining it with 2D planimetry could further enhance its accuracy.

## 1. Introduction

Transthoracic echocardiography (TTE) is the most common imaging modality used in the diagnosis and assessment of patients with valvular disorders, including mitral stenosis (MS) [1]. The mitral valve area (MVA) can be measured using an anatomic or hemodynamic assessment [2]. The direct anatomical measurement of the MVA can be performed using two-dimensional (2D) planimetry [3,4]. Other methods rely on assessing the hemodynamic effect of MS to derive the MVA. This includes the pressure half-time (PHT), continuity equation (CE), and proximal isovelocity surface area (PISA) methods [5,6,7]. The mean transmitral pressure gradient can also provide useful information on MS severity [8].

However, each of these measures have their limitations, and it is recommended that clinicians use a multi-parametric approach incorporating these echocardiographic methods and consider patients’ clinical statuses when managing patients with MS [9,10,11,12,13]. Our group recently described Yeo’s index, which is derived from the product of the mitral leaflet separation index (MLSI) and the dimensionless index (DI). We showed that Yeo’s index correlated well with other conventional echocardiographic measures of MS severity [14]. From a TEE perspective, TEE 3DMVA has been shown to demonstrate good agreement with MVA assessment by cardiac magnetic resonance imaging or invasive assessment [15,16,17]. With this in mind, there has been a growing acceptance that the three-dimensional assessment of the MVA (3DMVA) on TEE represents the new gold standard for the assessment of MS severity.

The objectives of this study are to validate Yeo’s index using TEE 3D MVA as a comparator and to compare the concordance of Yeo’s index and existing echocardiographic measures of MVA with TEE 3DMVA. We also aim to explore whether any combination of the TTE MVA measurement might give a better concordance with TEE 3DMVA.

## 2. Methodology

This was a retrospective chart review of an unselected sample of 111 cases of MS with varying severity who have undergone both TTE and TEE studies. This study conforms to the ethical guidelines of the 1975 Declaration of Helsinki and was approved by the National Healthcare Group Institutional Review Board (NHG DSRB 2021/00603). Echocardiographic recordings and data as well as relevant clinical information were obtained from electronic medical records and databases.

TEE 3DMVA was assessed using the 3D echocardiographic imaging platform (iE33; Philips Medical Systems, Andover, MA, USA) and a 3D TEE probe (X-9; Philips Medical Systems). TEE 3DMVA was measured using multiplanar reconstruction or direct planimetry of the narrowest mitral valve orifice. The TTE study with the shortest time interval to the TEE study was identified from the echocardiography database for the analysis of Yeo’s index and the measurement of the MVA using the 2D planimetry, PHT and CE methods. Yeo’s index was determined as the product of the MLSI and the DI. The maximal diastolic separation of the MV leaflet tips was assessed in the parasternal long-axis view and the apical four-chamber view, and the average was taken to be the MLSI [18]. A mean of 3 measurements was taken for patients with a sinus rhythm, while 5 measurements were taken in patients with atrial fibrillation. The DI was calculated by dividing the LVOT PW Doppler TVI by the MV CW Doppler TVI. An MVA measurement using 2D planimetry, PHT and CE assessments was performed in accordance with consensus guidelines [8]. These measurements were made by a single operator (TCY) who was blinded to the TEE 3DMVA and clinical data. In order to assess intra-observer reproducibility, 10 cases were selected at random, and measurements were first reperformed by the original operator (TCY) 4 months from the initial measurements. A blinded second operator (TL) was then tasked to reperform the measurements to assess inter-observer reproducibility. Intra- and inter-observer variability were assessed by intra-class correlation coefficients (ICCs) with 95% confidence intervals using SPSS 19 reliability analyses [19].

Continuous variables were expressed as the mean (±standard deviation), while categorical variables were expressed as a number (proportion). Each TTE measure of MS severity was analyzed separately, and cases where the clinical picture precluded its application were excluded. Linear regression analysis was first performed to determine the correlation between TEE 3D MVA and Yeo’s index as well as TEE 3D MVA and TTE measures of MVA by the planimetry, PHT and CE methods. Next, the various TTE MVA measures and Yeo’s index were analyzed for their concordance with TEE 3DMVA, which was taken to be the gold standard. This is presented in the form of the concordance statistic (*ρ*_c_) [20]. For the concordance analysis, we adopted pre-specified cut-offs that were derived from our previous study, where Yeo’s index values ≤ 0.147 cm and ≤0.260 cm corresponded to very severe MS (MVA ≤ 1.0 cm^2^) and severe MS (MVA ≤ 1.5 cm^2^), respectively [14]. For practicality reasons, we used a Yeo’s index of ≤0.15 cm for very severe MS instead. TEE 3DMVA was used to classify the cases of MS severity into non-severe (MVA > 1.5 cm^2^), severe MS (MVA > 1.0 cm^2^ but ≤1.5 cm^2^) and very severe MS (MVA ≤ 1.0 cm^2^), respectively. Finally, we sought to evaluate various combinations of the different methods of MVA assessment on TTE and Yeo’s index for their effectiveness in determining MS severity with reference to TEE 3DMVA. Statistical analysis was performed with IBM SPSS Statistics Version 26 (IBM Corp., Armonk, NY, USA) and MedCalc Statistical Software version 19.2.6 (MedCalc Software bv, Ostend, Belgium; https://www.medcalc.org; 2020). *p*-values were 2-sided and deemed significant if <0.05. Difference in Pearson’s correlation (r^2^) and concordance (*ρ_c_*) between different MVA measures and TEE 3DMVA were considered significant if there was no overlap in the 95% confidence intervals.

## 3. Results

The clinical characteristics, comorbidities and prescribed medical therapy of the cohort are shown in Table 1. The mean age of the cohort was 62.3 (±12.6) years, and 82 patients (73.9%) were female. A substantial 60 patients (54.1%) had atrial fibrillation at the time of the index echocardiogram, which was carried out during the initial diagnosis of MS, and 44 patients (39.6%) had hypertension, while 17 patients (15.3%) had ischemic heart disease. In terms of clinical presentation, 82 of the patients presented with dyspnea, and 10 had palpitations. There were 12 patients who presented for other reasons, but a subsequent evaluation including echocardiography revealed MS; this included 8 patients who presented with a stroke, 2 who presented with myocardial infarction and 2 who presented with infective endocarditis. There were seven patients who were asymptomatic and were referred for evaluation for murmurs at a clinical exam. In terms of medical therapy, 50.4% of the patients were undergoing oral anticoagulation therapy, while 55.0% of the patients were undergoing treatment with beta blockers.

The median time between TTE and TEE was 28 (interquartile range of 7–162, range of 1–352) days. Table 2 summarizes the distribution of MS severity according to the different methods of assessment. One case (0.9%) did not have suitable Doppler tracing for the measurement of PHT, while 18 cases (16.2%) had at least moderate mitral regurgitation (MR) or aortic regurgitation (AR) precluding the analysis using the CE. Yeo’s index and 2D planimetry can be measured in all patients. Most patients had severe or very severe MS according to the TEE 3D MVA assessment. A total of 44 patients (39.6%) had very severe MS and 58 patients (52.3%) had severe MS, while 9 (8.1%) had non-severe MS. The mean value for Yeo’s index was 0.19 (±0.11) cm. A total of 43 patients (38.8%) had a Yeo’s index of ≤0.15 cm, while 55 patients (49.5%) had a Yeo’s index of >0.15 cm but ≤0.26 cm, and 13 patients (11.7%) had a Yeo’s index of > 0.26 cm. The intra-observer and inter-observer variabilities for Yeo’s index, as assessed by the intraclass correlation coefficients (ICCs), were 0.981 (95% CI 0.90–0.99) and 0.96 (95% CI 0.86–0.98), respectively.

**Figure 1:**
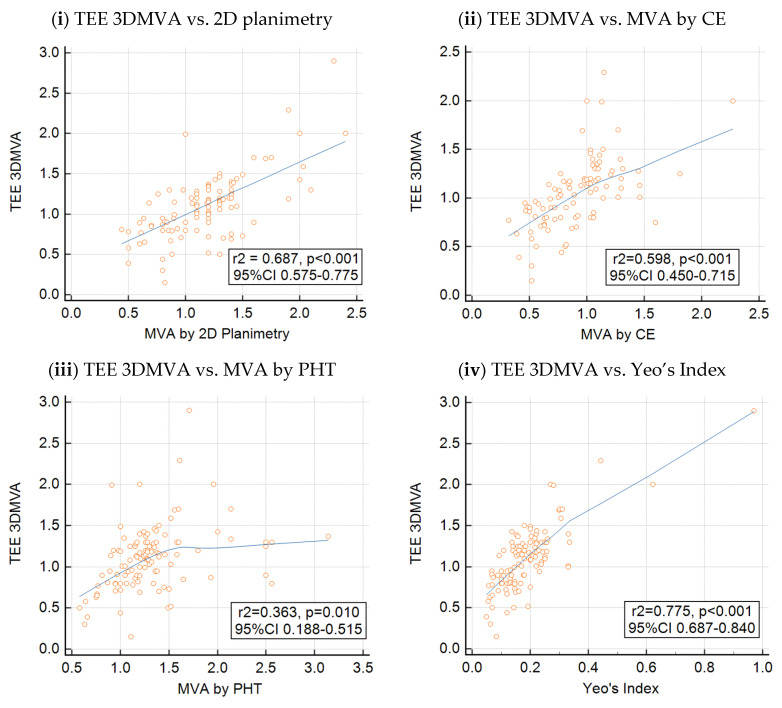
Peason’s correlation between TTE measures of MVA and TEE 3DMVA.

The Pearson’s correlation coefficients between TEE 3DMVA and Yeo’s index, MVA by 2D planimetry and the CE and PHT methods are shown in Figure 1, while the results of the concordance analysis carried out using TEE 3DMVA as the gold standard are shown in Table 3. Yeo’s index showed the best correlation with TEE 3DMVA (r^2^ = 0.775, *p* < 0.001) compared to PHT (r^2^ = 0.363, *p* = 0.01), CE (r^2^ = 0.598, *p* < 0.001) and 2D planimetry (r^2^ = 0.687, *p* < 0.001). Similarly, Yeo’s index values of ≤0.15 cm and ≤0.26 cm, corresponding to very severe MS (MVA ≤ 1.0 cm^2^) and severe MS (MVA ≤ 1.5 cm^2^), respectively, demonstrated the best concordance with TEE 3DMVA (*ρ*_c_ = 0.739) compared to PHT (*ρ*_c_ = 0.366), CE (*ρ*_c_ = 0.464) or 2D planimetry (*ρ*_c_ = 0.632) in the overall classification of MS severity. When tested separately for the classification for severe (MVA ≤ 1.5 cm^2^) and very severe MS (MVA ≤ 1.0 cm^2^), respectively, Yeo’s index retained the best concordance with TEE 3DMVA. A further subgroup analysis was performed to evaluate whether the underlying cardiac rhythm (AF vs. sinus rhythm) and the time interval between the TTE and TEE studies could have affected the results of the correlation and concordance analysis. As shown in Table 4, most of the echocardiographic parameters were unaffected by either the underlying rhythm or the time between studies. Only MVA by CE demonstrated a significantly improved performance in patients with a sinus rhythm compared to those in AF, while the other parameters were unaffected.

Table 5 shows the sensitivity and specificity of individual measures of MS severity compared to TEE 3DMVA. Yeo’s index demonstrated the best performance in classifying MS severity using TEE 3DMVA as a comparator for both severe and very severe MS. Table 6 shows the sensitivity, specificity and positive and negative predictive values of combined measures of MS severity using TEE 3DMVA as a comparator. The combination of Yeo’s index and 2D planimetry was highly associated with the presence or absence of very severe MS and severe MS in the TEE 3DMVA assessment. When both Yeo’s index and 2D planimetry suggested the presence of severe or very severe MS, the positive predictive value was high (AUC of 0.966 and PPV of 100.00% for severe MS; AUC of 0.864 and PPV of 85.71% for very severe MS). On the other hand, when both measures suggested the absence of very severe or severe MS, the negative predictive value was also high (AUC of 0.940 and NPV of 88.90% for severe MS; AUC of 0.831 and NPV of 88.71% for very severe MS).

## 4. Discussion

Despite advancements in transthoracic echocardiography, there remains no gold standard on TTE for the assessment of MS severity. Our group recently described Yeo’s index as a novel parameter that incorporates both anatomic (MLSI) and hemodynamic assessment (DI) and thus has the potential to be a useful addition to the armamentarium for MS assessment [14]. The MLSI is uniquely suited for the anatomic assessment of the severity of rheumatic MS, which presents with progressive leaflet thickening, commissural fusion and chordal thickening and fusion, leading to the characteristic funnel-shaped stenotic mitral valve [21]. The key limitation of TTE 2D planimetry lies in the difficulty of obtaining the narrowest flow-limiting orifice of the mitral valve on the parasternal short-axis view. Conversely, it is much easier to identify the narrowest point of the MV orifice on the parasternal long-axis and apical four-chamber views, as utilized in the MLSI. The mean transmitral gradient is commonly used as a hemodynamic measure of MS severity, but it is flow-dependent. The DI, on the other hand, is less flow-dependent as it is derived by dividing the LVOT PW Doppler TVI by the MV CW Doppler TVI. Incorporating the DI into Yeo’s index allows for the assessment of the hemodynamic significance of MS in addition to an anatomic assessment provided by the MLSI. Importantly, the measurements required to calculate Yeo’s index are all routine measurements during a TTE examination, and the improvements in diagnostic accuracy with Yeo’s index do not come at the expense of a longer scanning time. Furthermore, Yeo’s index can be measured and is feasible in all patients. This strengthens the role of Yeo’s index as a promising modification to the MLSI, which retains its practicality and ease of use while improving diagnostic performance in rheumatic MS.

### 4.1. Validation of Yeo’s Index against TEE 3DMVA

Our study confirmed that Yeo’s index, using the previously identified cut-offs of ≤0.15 cm and ≤0.26 cm, respectively, performed well in identifying patients with severe mitral stenosis. The performance of Yeo’s index was also not significantly affected by atrial fibrillation. Although there is a time interval between the TTE and TEE studies, it is not expected to affect the validity of the study as the progression of mitral stenosis is very slow [22]. Additionally, there was no significant difference in concordance between Yeo’s index and TEE 3DMVA in patients with a short time interval between the two studies (≤28 days) compared with those with a longer time interval. The use of TEE 3DMVA as a comparator is a strength of this study as TEE 3DMVA is now widely regarded as the gold standard measure of MVA [16].

### 4.2. Assessment of Correlation and Concordance of TTE MVA Assessments against TEE 3DMVA

Our study showed a good correlation between the different measures of MVA and TEE 3DMVA except for the MVA measurement using the PHT. Both Yeo’s index and 2D planimetry showed substantial concordance with TEE 3DMVA, whereas CE showed moderate agreement, and there was only slight agreement for the PHT. The poorer performance of the PHT in this study could be because our study included patients who are relatively older (mean age of 62.3 years) than historical cohort studies of rheumatic MS. The patients also had significant comorbidities of hypertension, diabetes mellitus and ischemic heart disease. Previous studies have shown that the accuracy of the PHT method is adversely affected by abnormal atrioventricular compliance, which is likely to be more prevalent in this cohort given their demographics [23,24]. There was no significant difference in the correlation between patients with atrial fibrillation and those with sinus rhythm for all measures of MVA. However, concordance between CE and TEE 3DMVA was significantly poorer in patients with atrial fibrillation compared to those with sinus rhythm. This is not unexpected as the main disadvantage of CE is the need for multiple measurements, hence increasing the possibility of error in measurement. This is expected to be worse in atrial fibrillation due to the irregularity in cardiac cycles.

### 4.3. Combination of 2D Planimetry and Yeo’s Index for Concordance with TEE 3DMVA

Because Yeo’s index and 2D planimetry had the best correlation and concordance with TEE 3DMVA, we further assessed whether their combination might result in the more accurate identification of patients with severe MS. When used together, 2D planimetry and Yeo’s index is useful for ruling in and ruling out severe MS. Should both 2D planimetry and Yeo’s index suggest that MS is not severe, it is highly unlikely that the patient has significant MS. On the other hand, should both measurements suggest the presence of severe or very severe MS, that assessment is also likely to be true. This can be useful in situations where there are discrepancies in the MVA derived by existing methods. The combination probably performed well because individually, they correlated best with TEE 3DMVA compared with the other methods of MVA assessment.

Hence, Yeo’s index can be a useful addition to the current armamentarium of echocardiographic parameters for the assessment of MS severity. It can be particularly useful in situations where there are discrepancies in MVA derived by existing methods. Importantly, we showed that the combination of Yeo’s index and MVA by 2D planimetry has a high diagnostic yield and is useful in ruling in or out severe MS in these situations.

## 5. Limitations

There are several limitations in our study. The findings of this study do not apply to degenerative MS, which is growing in prevalence, especially with an aging population. We did not validate Yeo’s index with invasive cardiac catheterization measures of MS severity. However, an invasive assessment of MS is not routinely performed currently, and TEE 3DMVA is the commonly accepted referenced standard. Our study predominantly included patients with severe or very severe MS, and our findings need further validation in patients without severe MS. The patient population is older than those used in previous studies, with a preponderance of females. This may limit the generalizability of the findings of this study to a younger and more balanced gender demographic. TTE and TEE were not performed in the same sitting, but we did not find a significant difference in correlation or concordance in patients with a short time interval between the two studies compared with those with a longer time interval. Finally, this was a single-center study that needs validation in other laboratories.

## 6. Conclusions

Yeo’s index performed well in identifying severe MS and demonstrated very good concordance with TEE 3DMVA compared to conventional echocardiographic measures of MS severity, including 2D planimetry, PHT and CE. It may be a useful adjunct to these existing measures of MS severity. When combined with 2D planimetry, its performance in identifying severe MS is further enhanced.

## Figures and Tables

**Table 1:** Clinical characteristics.

	Overall (*n* = 111)	Severe MS (*n* = 58)	Very Severe MS (*n* = 44)
Age at Diagnosis (years)	62.3 (±12.6)	63.14 (±11.96)	63.47 (±12.23)
Sex (female) (*n*, %)	82 (73.9%)	41 (70.7%)	35 (79.5%)
Ethnicity (%)			
Chinese	70 (63.1%)	37 (63.8%)	28 (63.6%)
Malay	16 (14.4%)	8 (13.8%)	6 (13.6%)
Indian	16 (14.4%)	9 (15.5%)	7 (15.9%)
Others	9 (8.1%)	4 (6.9%)	3 (6.8%)
Body Mass Index (kg/m^2^)	25.02 ± 5.99	25.18 (±5.63)	25.02 (±5.99)
Blood Pressure (mmHg)	128.6 (±21.5)/70.4 (±11.2)	131.84 (±21.86)/71.28 (±11.01)	126.27 (±21.07)/69.64 (±11.08)
Past Medical History			
Hypertension (%)	44 (39.6%)	19 (32.8%)	19 (43.2%)
Hyperlipidemia (%)	37 (33.3%)	20 (34.5%)	14 (31.8%)
Diabetes Mellitus (%)	18 (16.2%)	9 (15.5%)	9 (20.5%)
Ischemic Heart Disease (%)	17 (15.3%)	8 (13.8%)	7 (15.9%)
Chronic Kidney Disease (%)	6 (5.4%)	6 (10.3%)	0 (0.0%)
Atrial Fibrillation (%)	60 (54.1%)	32 (55.2%)	25 (56.8%)
Stroke (%)	15 (13.5%)	9 (13.8%)	7 (15.9%)
Medication Use			
Antiplatelet	25 (22.5%)	13 (22.4%)	12 (27.3%)
Oral Anticoagulation	56 (50.4%)	32 (55.0%)	22 (50.0%)
Beta Blocker	61 (55.0%)	30 (51.7%)	27 (61.4%)
ACE Inhibitor/ARB	15 (13.5%)	6 (10.3%)	6 (13.6%)
Calcium Channel Blocker	11 (9.9%)	6 (10.3%)	4 (9.1%)
Diuretics	28 (25.2%)	16 (27.6%)	11 (25.0%)

**Table 2:** Classifications of MS severity.

	3DMVA	2D Planimetry	PHT Method	CE Method	Yeo’s Index
Not Applicable Cases	0 (0.0%)	0 (0.0%)	1 (0.9%)	18 (16.2%)	0 (0.0%)
Mean Value	1.11 (±0.40)	1.18 (±0.38)	1.31 (±0.44)	0.92 (±0.32)	0.19 (±0.11)
Non-Severe MS (MVA > 1.5 cm^2^)	9 (8.1%)	12 (10.8%)	23 (20.9%)	3 (3.2%)	13 (11.7%)
Severe MS (1.0 < MVA ≤ 1.5 cm^2^)	58 (52.3%)	62 (55.9%)	62 (56.4%)	35 (37.6%)	55 (49.5%)
Very Severe MS (MVA ≤ 1.0 cm^2^)	44 (39.6%)	37 (33.3%)	25 (22.7%)	55 (59.1%)	43 (38.8%)

**Table 3:** Concordance of echocardiographic measures compared to TEE 3DMVA.

	MS Severity in Reference to the Following:		
	3DMVA	3DMVA ≤ 1.0 cm^2^	3DMVA ≤ 1.5 cm^2^
2D Planimetry	*ρ*_c_ = 0.661	*ρ*_c_ = 0.632	*ρ*_c_ = 0.738
Continuity Eq	*ρ*_c_ = 0.464	*ρ*_c_ = 0.514	*ρ*_c_ = 0.187
PHT	*ρ*_c_ = 0.366	*ρ*_c_ = 0.367	*ρ*_c_ = 0.363
Yeo’s Index	*ρ*_c_ = 0.739	*ρ*_c_ = 0.717	*ρ*_c_ = 0.799

The *ρ*_c_ can take values from −1 to 1 and is interpreted somewhat arbitrarily as follows: 0 = agreement equivalent to chance; 0.10–0.20 = slight agreement; 0.21–0.40 = fair agreement; 0.41–0.60 = moderate agreement; 0.61–0.80 = substantial agreement; 0.81–0.99 = near-perfect agreement; and 1.00 = perfect agreement.

**Table 4:** Subgroup analysis stratified by AF and TEE/TTE duration.

			2D Planimetry	MVA by CE	MVA by PHT	Yeo’s Index
Correlation to TEE3DMVA	By AF Status	AF	r^2^ = 0.583, *p* < 0.001 (0.387–0.729)	r^2^ = 0.529, *p* < 0.001 (0.296–0.702)	r^2^ = 0.322, *p* = 0.012 (0.075–0.533)	r^2^ = 0.666, *p* < 0.001 (0.496–0.787)
		Sinus Rhythm	r^2^ = 0.784, *p* < 0.001 (0.649–0.872)	r^2^ = 0.685, *p* < 0.001 (0.481–0.818)	r^2^ = 0.377, *p* = 0.007 (0.111–0.593)	r^2^ = 0.847, *p* < 0.001 (0.745–0.910)
	By Interval Duration	Duration < 28 days	r^2^ = 0.751, *p* < 0.001 (0.604–0.849)	r^2^ = 0.655, *p* < 0.001 (0.438–0.780)	r^2^ = 0.362, *p* = 0.008 (0.102–0.576)	r^2^ = 0.812, *p* < 0.001 (0.694–0.888)
		Duration > 28 days	r^2^ = 0.641, *p* < 0.001 (0.458–0.771)	r^2^ = 0.568, *p* < 0.001 (0.347–0.730)	r^2^ = 0.368, *p* = 0.005 (0.118–0.573)	r^2^ = 0.731, *p* < 0.001 (0.583–0.832)
Concordance to TEE 3DMVA	By AF Status	AF	*ρ*_c_ = 0.600 (0.413–0.739)	*ρ*_c_ = 0.264 (0.008–0.487)	*ρ*_c_ = 0.303 (0.084–0.496)	*ρ*_c_ = 0.692 (0.535–0.802)
		Sinus Rhythm	*ρ*_c_ = 0.744 (0.593–0.844)	*ρ*_c_ = 0.690 (0.498–0.817)	*ρ*_c_ = 0.457 (0.233–0.635)	*ρ*_c_ = 0.841 (0.737–0.906)
	By Interval Duration	Duration < 28 days	*ρ*_c_ = 0.679 (0.504–0.800)	*ρ*_c_ = 0.527 (0.284–0.706)	*ρ*_c_ = 0.396 (0.156–0.592)	*ρ*_c_ = 0.785 (0.657–0.870)
		Duration > 28 days	*ρ*_c_ = 0.666 (0.497–0.786)	*ρ*_c_ = 0.391 (0.141–0.594)	*ρ*_c_ = 0.385 (0.181–0.557)	*ρ*_c_ = 0.742 (0.601–0.838)

**Table 5:** Sensitivity and specificity of individual measures of MS severity compared to 3D planimetry on transesophageal echocardiogram.

**(a) For Very Severe MS (MVA ≤ 1.0 cm^2^)**				
	**AUC**	**95% CI**	**Sensitivity**	**Specificity**
MVA ≤ 1.0 cm^2^ by 2D planimetry	0.807	0.698–0.872	70.45%	91.04%
MVA ≤ 1.0 cm^2^ by CE	0.764	0.660–0.843	88.10%	64.71%
MVA ≤ 1.0 cm^2^ by PHT	0.670	0.571–0.770	43.18%	90.91%
Yeo’s Index ≤ 0.15 cm	0.857	0.778–0.927	81.82%	89.55%
	MVA ≤ 1.0 cm^2^ by 2D planimetry	MVA ≤ 1.0 cm^2^ by CE	MVA ≤ 1.0 cm^2^ by PHT	Yeo’s Index ≤ 0.15 cm
MVA ≤ 1.0 cm^2^ by 2D planimetry	-	*p* = 0.563	*p* = 0.013	*p* = 0.086
MVA ≤ 1.0 cm^2^ by CE		-	*p* = 0.152	*p* = 0.037
MVA ≤ 1.0 cm^2^ by PHT			-	*p* < 0.001
Yeo’s Index ≤ 0.15 cm				-
**(b) For Severe MS (MVA ≤ 1.5 cm^2^)**				
	**AUC**	**95% CI**	**Sensitivity**	**Specificity**
MVA ≤ 1.5 cm^2^ by 2D planimetry	0.925	0.826–0.956	96.08%	88.89%
MVA ≤ 1.5 cm^2^ by CE	0.572	0.464–0.674	97.70%	16.67%
MVA ≤ 1.5 cm^2^ by PHT	0.810	0.664–0.846	84.16%	77.78%
Yeo’s Index ≤ 0.26 cm	0.980	0.940–1.000	96.08%	100.00%
	MVA ≤ 1.5 cm^2^ by 2D planimetry	MVA ≤ 1.5 cm^2^ by CE	MVA ≤ 1.5 cm^2^ by PHT	Yeo’s Index ≤ 0.26 cm
MVA ≤ 1.5 cm^2^ by 2D planimetry	-	*p* = 0.002	*p* = 0.009	*p* = 0.322
MVA ≤ 1.5 cm^2^ by CE		-	*p* = 0.091	*p* < 0.001
MVA ≤ 1.5 cm^2^ by PHT			-	*p* = 0.037
Yeo’s Index ≤ 0.26 cm				-

**Table 6:** Sensitivity, specificity and positive and negative predictive values of combined measures of MS severity compared to 3D planimetry on transesophageal echocardiogram.

**(a) For Very Severe MS (MVA ≤ 1.0 cm^2^)**					
	**AUC**	**Sensitivity**	**Specificity**	**PPV**	**NPV**
Very severe MS by 2D planimetry or Yeo’s index	0.831	84.09%	82.09%	75.51%	88.71%
Very severe MS by 2D planimetry and Yeo’s index	0.864	81.82%	91.04%	85.71%	88.41%
**(b) For Severe MS (MVA ≤ 1.5 cm^2^)**					
	**AUC**	**Sensitivity**	**Specificity**	**PPV**	**NPV**
Severe MS by 2D planimetry or Yeo’s index	0.940	99.02%	88.89%	99.02%	88.90%
Severe MS by 2D planimetry and Yeo’s index	0.966	93.14%	100.00%	100%	56.27%

## Data Availability

The data presented in this study are available on request from the corresponding author.
